# Complexation with Random Methyl-β-Cyclodextrin and (2-Hydroxypropyl)-β-Cyclodextrin Promotes Chrysin Effect and Potential for Liver Fibrosis Therapy

**DOI:** 10.3390/ma13215003

**Published:** 2020-11-06

**Authors:** Simona-Rebeca Ignat, Sorina Dinescu, Judit Váradi, Ferenc Fenyvesi, Thi Le Phuong Nguyen, Alina Ciceu, Anca Hermenean, Marieta Costache

**Affiliations:** 1 Department of Biochemistry and Molecular Biology, Faculty of Biology, University of Bucharest, Splaiul Independentei 91-95, 050095 Bucharest, Romania; simona.ignat@unibuc.ro (S.-R.I.); alinaciceu80@gmail.com (A.C.); anca.hermenean@gmail.com (A.H.); marieta.costache@bio.unibuc.ro (M.C.); 2 Research Institute of the University of Bucharest, Splaiul Independentei 91-95, 050663 Bucharest, Romania; 3 Department of Pharmaceutical Technology, Faculty of Pharmacy, University of Debrecen, Nagyerdei St. 98, H-4032 Debrecen, Hungary; varadi.judit@pharm.unideb.hu (J.V.); fenyvesi.ferenc@pharm.unideb.hu (F.F.); nguyen.thi.le.phuong@pharm.unideb.hu (T.L.P.N.); 4 Doctoral School of Pharmaceutical Sciences, University of Debrecen, Nagyerdei St. 98, H-4032 Debrecen, Hungary; 5 “Aurel Ardelean” Institute of Life Sciences, Vasile Goldis Western University of Arad, 86 Revolutiei, 310025 Arad, Romania

**Keywords:** chrysin, cyclodextrins, liver fibrosis, hepatic stellate cells, biocompatibility, anti-inflammatory, antioxidant, anti-fibrotic

## Abstract

Liver fibrosis results from chronic liver injury and is characterized by the accumulation of extracellular matrix in excess driven by hepatic stellate cells (HSCs) activation. Chrysin (CHR) is a natural flavonoid that is limited by its low solubility to exert its anti-inflammatory, antioxidant and anti-fibrotic properties. The aim of this study was to investigate the biocompatibility of CHR complexes with two cyclodextrins (CDs)-(2-hydroxypropyl)-β-cyclodextrin (HPBCD) and random methyl-β-cyclodextrin (RAMEB), and their potential to induce anti-inflammatory, antioxidant and anti-fibrotic effects. Biocompatibility of the complexes was evaluated on Huh7 and LX2 cell lines: MTT and Live/Dead tests indicated the cell viability and an LDH test showed the cytotoxicity. Immunohistochemical staining of Nuclear Factor Kappa B (NF-κB) nuclear translocation was performed to evaluate the anti-inflammatory effect of the complexes. Oxygen Radical Absorbance assay, Superoxide Dismutase activity and Glutathione Peroxidase (GPx) assays indicated the antioxidant properties of the chrysin complexes. Finally, the complexes’ anti-fibrotic potential was evaluated at the protein and gene level of *α-sma*. In HSCs, CDs induced higher cytotoxicity correlated with lower cell viability than CHR–CD. The 1:1 CHR–RAMEB pretreatment avoided p65 translocation. The 1:2 CHR–RAMEB complex increased ORAC values, improved SOD activity and produced the highest stimulation of GPx activity. CHR–RAMEB reduced *α-sma* expression at lower concentration than CHR–HPBCD, proving to be more efficient. In conclusion, both CHR–CD complexes proved to be biocompatible, but CHR–RAMEB showed improved anti-inflammatory, antioxidant and anti-fibrotic effects that could recommend its further use in liver fibrosis treatment.

## 1. Introduction

Liver fibrosis is generated in the presence of a chronic liver injury that induces hepatic stellate cells (HSCs) activation and excessive extracellular matrix (ECM) accumulation [1,2]. If untreated, liver fibrosis can progress to cirrhosis or hepatocellular carcinoma, which represent irreversible serious health issues affecting many people worldwide [3]. At the center of liver fibrosis progression are the activated HSCs that acquire a myofibroblast-*like* phenotype, expressing alpha smooth muscle actin (*α-sma*) and secreting collagen type I (col I), the main component of accumulating ECM. These cells are responsive to multiple growth factors, such as transforming growth factor β (TGF-β) and platelet-derived growth factor (PDGF) that induce their proliferation and fibrogenic behavior. Furthermore, activated HSCs also produce TGF-β themselves, creating a positive activating loop [3,4]. 

As liver fibrosis is a reversible stage in liver disease progression, many treatments target it to prevent or attenuate its progress. Some of them target activated HSCs to either reverse to a latent state, or to undergo apoptosis. To date, there is no standard treatment to induce liver fibrosis reversion, and new potential therapies are currently searched and tested. Natural compounds such as helioxanthin, silymarin, silybinin, curcumin were evaluated for their anti-fibrotic potential [5,6,7]. Such a treatment could be based on chrysin (5,7-dihydroxyflavone) (CHR), a bioactive flavonoid from propolis, honey and plant extracts that proves to have multiple valuable properties: anti-inflammatory, antioxidant, anti-bacterial, anti-tumor, hepatoprotective [8,9,10,11,12,13]. Moreover, the anti-fibrotic potential in liver fibrosis was investigated and confirmed in recent studies [9,14]. CHR was shown to modulate ECM deposition by restoring the balance between tissue inhibitors of metalloproteinases (TIMPs) and matrix metalloproteinases (MMPs). In addition, it acts on the TGF-β pathway by reducing the *α-sma* and TGF-b1/Smad 2 and 3 expression.

However, the efficiency of this potential treatment is limited by its low solubility and reduced absorption [15]. Therefore, CHR bioavailability should be improved in order to allow CHR to fully exhibit its potential in attenuating liver fibrosis. Such a result could be obtained by complexation with cyclodextrins (CDs) [16,17]. CDs are cyclic oligosaccharides derived from starch degradation and frequently used as drug solubilizers [18,19]. Random methyl-β-cyclodextrin (RAMEB) and (2-hydroxypropyl)-β-cyclodextrin (HPBCD) are two types of CDs that induce low toxicity, and original complexes with CHR were developed and characterized in a previous study of our group [20]. We found that the complexation of CHR with cyclodextrins improved its solubility and permeability through Caco-2 monolayers. In the present study, we aimed to evaluate the in vitro biocompatibility of these newly developed complexes and to investigate in depth the anti-inflammatory, antioxidant and anti-fibrotic effects of these complexes in order to validate them as a potential treatment in liver fibrosis. Biocompatibility was analyzed comparatively, on hepatocellular carcinoma cell line Huh7 and on hepatic stellate cell line LX2, while the anti-inflammatory and antioxidant potential was assessed on intestinal Caco-2 cells and anti-fibrotic potential on LX2 cells. This study completes the analysis and biological characterization of CHR–RAMEB and CHR–HPBCD complexes previously synthesized and discussed by our group [15] and offers a functional and accessible alternative therapy in liver fibrosis for further clinical use.

## 2. Materials and Methods

### 2.1. Preparation of Chrysin–Cyclodextrin Complexes and Dynamic Light Scattering and Zeta-Potential Measurement

(2-hydroxypropyl)-β-cyclodextrin (HPBCD) (degree of substitution (DS) ~ 4.5; MW = 1396) and random methyl-β-cyclodextrin (RAMEB) (DS ~ 12; MW = 1303) were the product of Cyclolab Ltd. (Budapest, Hungary). CHR–RAMEB and CHR–HPBCD complexes with 1:1 and 1:2 molar ratios were synthesized as previously described [20]. Complexes were solubilized in phosphate-buffered saline solution (PBS) to stock solutions of 1mM, and were further diluted in cell media 10–300 µM.

Saturated solutions of CHR–cyclodextrin complexes were prepared with PBS by mixing excess amounts of complexes for 24 h at room temperature. The solutions were centrifuged at 10,000 rpm for 6 min and the clear supernatants were used for the measurements applying Zetasizer Nano ZSP (Malvern Panalytical Ltd., Malvern, UK).

### 2.2. Cell Cultures

Cells derived from human hepatocellular carcinoma (Huh7) were propagated in culture with Dulbecco’s Modified Eagle Medium (DMEM) (Sigma-Aldrich, Saint Louis, MO, USA) media supplemented with 10% fetal bovine serum (FBS, Gibco) and 1% antibiotic antimycotic (ABAM, Sigma-Aldrich). In addition, hepatic stellate cells from LX2 cell line were kindly provided by Dr. Pau Sancho-Bru with permission from Dr. Scott Friedman (Icahn School of Medicine at Mount Sinai, New York, NY, USA). HSCs were maintained in DMEM media supplemented with 2% SFB and 1% ABAM. Cell cultures were incubated at 37 °C, 5% CO_2_ with humidity. HSCs were activated with 10 ng/mL TGF-β1 for 24 h (Cell Signaling). Human Caco-2 intestinal epithelial cells were obtained from the European Collection of Cell Cultures (ECACC, UK) and grown routinely in Dulbecco’s Minimum Essential Medium (DMEM), supplemented with 10% fetal bovine serum (FBS), 1% non-essential amino acid and 1% penicillin–streptomycin solution.

### 2.3. Biocompatibility Evaluation

Cells were cultured in 48 well plates at 4 × 10^4^ cells/cm^2^ (Huh7) and 1.5 × 10^4^ cells/cm^2^ (LX2). After 24 h, cells were put in contact with the complexes at concentrations of 10–300 µM for another 48 h. Biocompatibility of the complexes was evaluated by quantitative (MTT and LDH tests, Sigma-Aldrich) and qualitative tests (LiveDead Assay, ThermoScientific, Waltham, MA, USA). By MTT, cell viability was evaluated in the presence of the complexes. Cells were washed with phosphate buffered saline (PBS, Sigma-Aldrich), and maintained with 3-(4,5-Dimethylthiazol-2-yl)-2,5-diphenyltetrazolium bromide 1 mg/mL for 4 h. Metabolic active cells turned the compound to formazan crystals, soluble in isopropanol and the absorbance was measured at 550 nm by spectrophotometry. The values for the absorbance were proportional to the number of live cells in the wells. LDH assay indicated the cytotoxicity levels induced by the complexes on the cells they were exposed to. Cell media was collected and following the kit instructions, the mix was added for 15 min RT in a dark room. The absorbance was measured at 490 nm by spectrophotometry. The absorbance values correspond to the number of dead cells in the wells. For the LiveDead Assay, live cells were stained with calcein AM (green) and nuclei of the dead cells with ethidium homodimer (red). Cells were visualized by fluorescence microscopy (IX-73 Olympus, Tokyo, Japan).

### 2.4. Immunohistochemical Staining and Analysis of Nuclear Factor Kappa B (NF-κB) Nuclear Translocation

Caco-2 cells were seeded onto sterile uncoated microscope cover slides at a density of 5 × 10^4^ cells/slide and cultured for 6 days. Caco-2 cultures were divided into groups according to the stimulant(s) used. Following 6 days of culture in DMEM, the medium was renewed, and cells were pretreated with different CHR–CD complexes at 25 µM concentration or with saturated chrysin aqueous solution for 60 min. After that, cells were treated with 50 ng/mL Tumor Necrosis Factor-α (TNF-α) for 30 min at 37 °C. Then, each slide was fixed with ice-cold methanol-acetone (1:1) and non-specific antibody binding sites were blocked with FBS for 15 min. Primary labelling of the NF-κB p65 subunit was conducted using 2 μg/mL rabbit anti-human p65 antibody (Santa Cruz Biotechnology) for 60 min at 37 °C. The samples were washed three times with Hank’s Balanced Salt Solution (HBSS) and secondary staining with Alexa Fluor 488-conjugated goat-anti-rabbit IgG was applied (Thermo Fisher Scientific, Waltham, MA, USA). Samples were washed four times with HBSS and cell nuclei were stained with Hoechst 33342 (0.1 µg/mL) for 15 min at room temperature. After washing with HBSS, the cover slides were mounted on glass microscopy slides and observed by a Zeiss Axio Scope A1 fluorescent microscope (HBO 100 lamp) (Carl Zeiss Microimaging GmbH, Göttingen, Germany). Images were analyzed with ZEN software, and the ratio of nuclear and perinuclear fluorescence intensities of the cells was calculated.

### 2.5. Superoxide Dismutase (SOD) Assay

The SOD Assay kit (Cayman Chemical, Ann Arbor, MI, USA) was used for measuring SOD enzymes. Caco-2 cells were cultured in 12-well plates at the density of 4 × 10^5^ cells per well and then pretreated with 25 µM CHR–CD complexes for 1 h. After that, samples were treated with 50 ng/mL TNF-α for the induction of O^2−^ or 1 h and then the wells were washed with PBS. Caco-2 cells were collected by adding 500 µL trypsin to each well and incubating at 37 °C for 10 min. Cells were placed into Eppendorf tubes on ice, the volumes were completed with 4 mL PBS and then they were centrifuged at 500× *g* for 10 min at 4 °C. The supernatants were discarded. Cell pellets were re-suspended in 1.2 mL ice-cold PBS, centrifuged at 250× *g* for 10 min at 4 °C and the supernatant was discarded. Cell pellet was homogenized in 0.5–1 mL PBS per 100 mg of cells and then centrifuged at 10,000× *g* for 15 min at 4 °C. The supernatant was collected for experiments. The preparation of standard solutions and reagents as well as the procedure for assay were done by the protocol provided with the kit. The antioxidant enzyme activity was measured by inhibiting of the dye reduction as the result of oxygen generation by xanthine oxidase. The samples were measured at 450 nm with a FLUOstar OPTIMA microplate reader (BMG LABTECH, Offenburg, Germany), and the results were expressed as U/mL.

### 2.6. Glutathione Peroxidase Assay

The seeded Caco-2 cells at the density of 4 × 10^5^ cells per well were treated with 25 µM Chrysin–CD complexes for 1 h. Cell lysates were produced by removing medium then washing cells with 5 mL Hank’s solution for two times. After that, cells were collected, centrifuged (1000–2000× *g* for 10 min at 4 °C) and homogenized in cold buffer then centrifuged again (10,000× *g* for 15 min at 4 °C). The supernatant was removed for assay and stored on ice. Experimental reagents such as GPx assay and GPx sample buffer, GPx (control), GPx Co-substrate mixture, GPx cumene hyperoxide and NADPH were prepared according to protocol of Glutathione Peroxidase assay kit (Cayman Chemical, Ann Arbor, MI, USA).

The depletion of NADPH at 340 nm was recorded with a FLUOstar OPTIMA microplate reader (BMG LABTECH, Offenburg, Germany) once every minute for 7 cycles at 25 °C. The enzyme activity then was calculated as nmol/min/mg protein using the NADPH extinction coefficient of 0.00373 µM^−1^.

### 2.7. Oxygen Radical Absorbance Assay (ORAC Assay)

The free-radical generator 2,2′-Azobis(2-amidinopropane) dihydrochloride (AAPH) was prepared by mixing with phosphate buffer KH_2_PO_4_-K_2_PO_4_ (75 mM, PH = 7) to obtain an 18.75 mM concentration solution. The fluorescein solution was applied at 0.6 µM final concentration. Cyclodextrins and CHR–CD complexes were dissolved at a concentration of 25 µM; chrysin aqueous solution was prepared by saturation in water.

The reaction was initiated by immediately adding AAPH to samples and the signal was measured for 1 h at every 2 min with a FLUOstar OPTIMA microplate reader (BMG LABTECH, Offenburg, Germany, excitation λ = 485 nm, emission λ = 528 nm). The free-radical generator produced peroxyl radicals, resulting in loss of fluorescence due to the damage of fluorescein molecules. The intensity of fluorescence vs. time was plotted and the area under the curve of chrysin and complexes were determined. The ORAC fluorescence indexes were calculated for different C–CD and cyclodextrin derivates. The difference of ORAC indexes for C–CD and same cyclodextrin derivate was compared to the ORAC index of chrysin.

### 2.8. Antifibrotic Effect of the Complexes on LX2 Cell Line

To evaluate the antifibrotic potential of the complexes, HSCs were cultured in 48 well plates at 1.5 × 10^4^ cells/cm^2^. Following TGF-β activation, cells were put in contact with the complexes for a further 48 h. The level of activation marker *α-sma* was evaluated both at the gene (qPCR) and protein (immunofluorescence, IF) levels. For the gene expression study, total RNA was extracted with TRIzol Reagent (ThermoScientific, Waltham, MA, USA). RNA concentration and purity were determined on a NanoDrop 8000 Spectrophotometer (Thermo Fisher Scientific, Waltham, MA, USA) and RNA integrity was assessed on a BioAnalyzer 2100 (Agilent Technologies). Using the iScript cDNA synthesis kit (BioRad), cDNA was obtained and amplified on a Veriti 96-Well Thermal Cycler (Applied Biosystems). For Real Time PCR, the SYB Select Master Mix (ThermoScientific, Waltham, MA, USA) and ViiA 7 Real-Time PCR System (ThermoScientific, Waltham, MA, USA) were used. Samples were tested in triplicate and the glyceraldehyde 3-phosphate dehydrogenase (*gapdh*) gene was used as reference. Primers used were: *α-sma* F 5′ TTCGCATCAAGGCCCAAGAA 3′, *α-sma* R 5′ GTCCCGGGGATAGGCAAAG 3′, *gapdh* F 5′ GAGTCAACGGGGTCGT 3′, *gapdh* R 5′ TTGATTTTGGATCTCG 3′.

For the evaluation of *α-sma* expression, cells were fixed with 4% paraformaldehyde solution for 20 min, followed by permeabilization for 1 h with 2% bovine serum albumin (BSA) solution with 0.1% Triton X100. Overnight at 4 °C, samples were incubated with antibody anti-*α-sma* (sc-130616, Santa Cruz Biotechnology), and then incubated for 1 h with antibody anti-mouse conjugated with fluorescein isothiocyanate (FITC) (Santa Cruz Biotechnology). To visualize the nuclei, cells were stained with Hoechst 33342 (Sigma-Aldrich). Cells were visualized by fluorescence microscopy (IX-73 Olympus).

All results were statistically analyzed with GraphPad Prism 6.0, one-way ANOVA and Bonferroni correction; *p*-values < 0.05 were considered statistically significant in accordance with the 95% confidence interval established for the analysis.

## 3. Results

### 3.1. Characteristics of Chrysin–Cyclodextrin Complexes Measured by Dynamic Light Scattering

The characteristics of CHR–RAMEB and CHR–HPBCD complexes were measured by dynamic light scattering and the results are presented in Table 1. CHR–RAMEB and CHR–HPBCD complexes have similar hydrodynamic diameters, which correspond to the size of the cyclodextrin molecules (peak 1). A larger particle population (peak 2) can be found in the samples according to the intensity distributions. Interestingly, CHR–RAMEB complexes have higher peak area intensity values in the case of smaller species (peak 1 area intensity %) compared to CHR–HPBCD complexes, which shows the better stabilization properties of RAMEB in water. This is in accordance with our previous solubility results, where 1:2 CHR–RAMEB complex showed the best solubility in water. CHR–HPBCD complexes have species with larger hydrodynamic diameters and higher peak area intensity values (Peak 2 average area intensity %), indicating larger light scattering intensity, which can explain the tendency of CHR–HPBCD complexes for aggregation. There were no significant differences among the zeta-potentials of the CHR–cyclodextrin complexes.

### 3.2. Biocompatibility of the Complexes

#### 3.2.1. MTT Test

**Figure 1:**
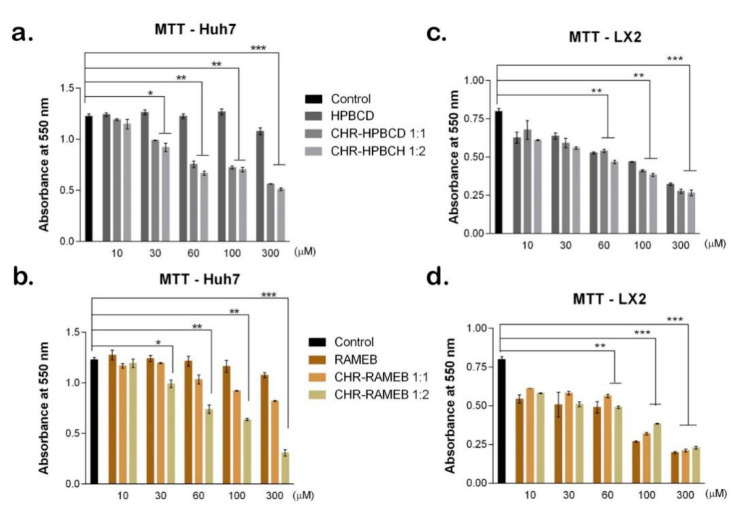
Huh7 and LX2 viability in contact with CD–CHR complexes evaluated by quantitative MTT test. Statistical significance: * *p* < 0.05; ** *p* < 0.01; *** *p* < 0.001. (**a**) Huh7 viability in contact with HPBCD complexes; (**b**) Huh7 viability in contact with RAMEB complexes; (**c**) LX2 viability in contact with HPBCD complexes; (**d**) LX2 viability in contact with RAMEB complexes.

Cell viability was evaluated by MTT test both on Huh7 and LX2 exposed to CHR complexes (1:1 and 1:2), as well as CDs alone (Figure 1). Both CDs included in this study, HPBCD and RAMEB, showed little influence on Huh7 viability, even at high concentrations (300 µM), as Huh7 is a cell line derived from carcinoma, and as such, is more resilient. However, when CHR was complexed with the CDs, the cell viability statistically significantly dropped compared to control, starting from 30 µM (*p* < 0.05), suggesting the efficient release of CHR from the complexes. As the concentration was increased, the cell viability was statistically significantly reduced starting from 30 µM (*p* < 0.05) 1:1 and 1:2 CHR–HPBCD and only 1:2 CHR–RAMEB. Both CHR–HPBCD complexes (1:1 and 1:2) induced similar responses in Huh7 viability all throughout the five concentrations tested (Figure 1a). In contrast, CHR from 1:2 CHR–RAMEB was released more efficiently compared to 1:1 complexes, as the cell viability for 1:2 CHR–RAMEB 30 µM was similar to the cell viability for 1:1 CHR–RAMEB 60 µM. The same situation was observed for cell viability in contact with 1:2 CHR–RAMEB 60 µM and 1:1 CHR–RAMEB 300 µM. This suggests the 1:2 RAMEB complexes could increase the CHR bioavailability at a smaller concentration than needed for 1:1 complexes to obtain the same cellular response. The Huh7 response to CHR–HPBCD complexes 60 and 100 µM was similar between them and the cell viability was significantly reduced (*p* < 0.01). As for RAMEB complexes 60 and 100 µM, the same behavior was observed as they induced similar cell viability levels for each concentration, but the cell viability in contact with 1:1 complexes, even though was reduced compared to 30 µM, was still higher than for 1:2 complexes. The most reduced Huh7 viability compared to control was in contact with CHR–HPBCD 300 µM (*p* < 0.01) (cell viability reduced ~50%) and also for CHR–RAMEB 300 µM, but only the 1:2 complex (cell viability reduced ~80%), suggesting the higher efficiency of 1:2 RAMEB complex in releasing CHR.

HSCs were also exposed to CDs and CHR complexes and cell viability was measured (Figure 1c,d). In contrast to Huh7 response, HSCs viability was affected in similar levels by exposure to CDs and both types of CHR complexes, for each concentration. There are no statistically significant differences in cell viability levels between the three conditions at each concentration. This suggests that the obvious decrease in cell viability compared to HSC control, could be induced by the CDs alone, and not the CHR presence in the complex. Moreover, CHR from RAMEB complexes (Figure 1d) improved cell viability compared to RAMEB, for all concentrations tested, suggesting the positive effect of CHR in the complex on cell viability. Starting from 10 µM, both types of CDs and their CHR complexes reduced HSCs viability as the concentration was increased up to 300 µM, with statistically significant differences compared to control. Most significantly, HSCs viability was reduced compared to control, when exposed to 60 µM HPBCD complexes (*p* < 0.01) (~35%) and to 100 µM RAMEB complexes (*p* < 0.001) (~45%). Cell viability levels in the presence of 300 µM of both types of complexes were significantly affected compared to control and not recommended for use (*p* < 0.001).

#### 3.2.2. LDH Test

**Figure 2:**
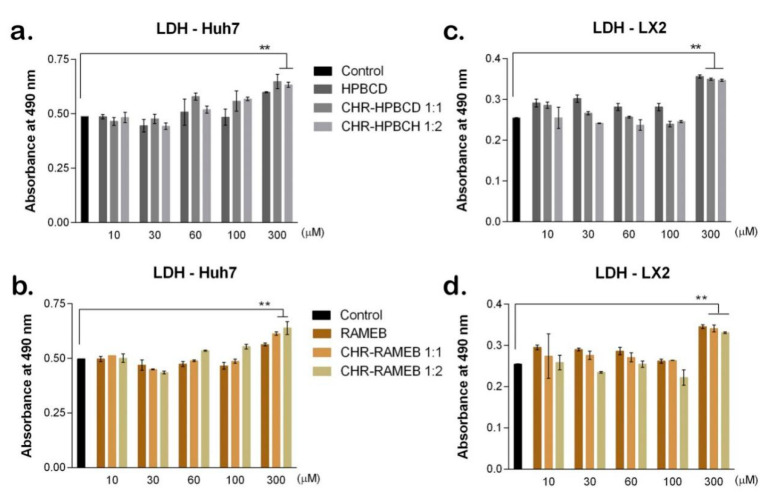
CD–CHR cytotoxicity induced on Huh7 and LX2, evaluated by quantitative LDH test. Statistical significance: * *p* < 0.05; ** *p* < 0.01; (**a**) Cytotoxicity induced by HPBCD complexes on Huh7; (**b**) Cytotoxicity induced by HPBCD complexes on LX2; (**c**) Cytotoxicity induced by RAMEB complexes on Huh7; (**d**) Cytotoxicity induced by RAMEB complexes on LX2.

The LDH test showed cytotoxicity levels induced by exposure to CDs and CHR complexes on Huh7 and LX2 cells (Figure 2). The cytotoxicity levels presented in Figure 2 showed similar profiles in all four LDH plots. Complexes of 10 and 30 µM did not induce higher levels of cytotoxicity on Huh7 compared to control. However, starting with 60 µM, complexes with CHR induced higher cytotoxicty compared to control and to CDs alone, suggesting that CHR was successfully released from the CD complexes and induced tumor cells to die (Figure 2a,c). Both 300 µM HPBCD and RAMEB complexes with CHR induced statistically significant higher levels for cell death compared to control, suggesting the increased level of cytotoxicity induced at this high concentration. As for the impact on LX2, both types of CDs induced higher levels of cytotoxicity compared to control, starting from 10 µM, in accordance with the reduced cell viability in the same conditions evaluated by MTT test. Compared to the level of cytotoxicity determined by CDs alone, for each concentration, the CD–CHR complexes induced much lower levels, suggesting the positive effect of CHR in the complex. Complexes induced statistically significant high levels (*p* > 0.01) of cytotoxicity at 300 µM concentration compared to control, suggesting the harmful effect the complexes could induce in such high concentrations.

#### 3.2.3. LiveDead Assay

**Figure 3:**
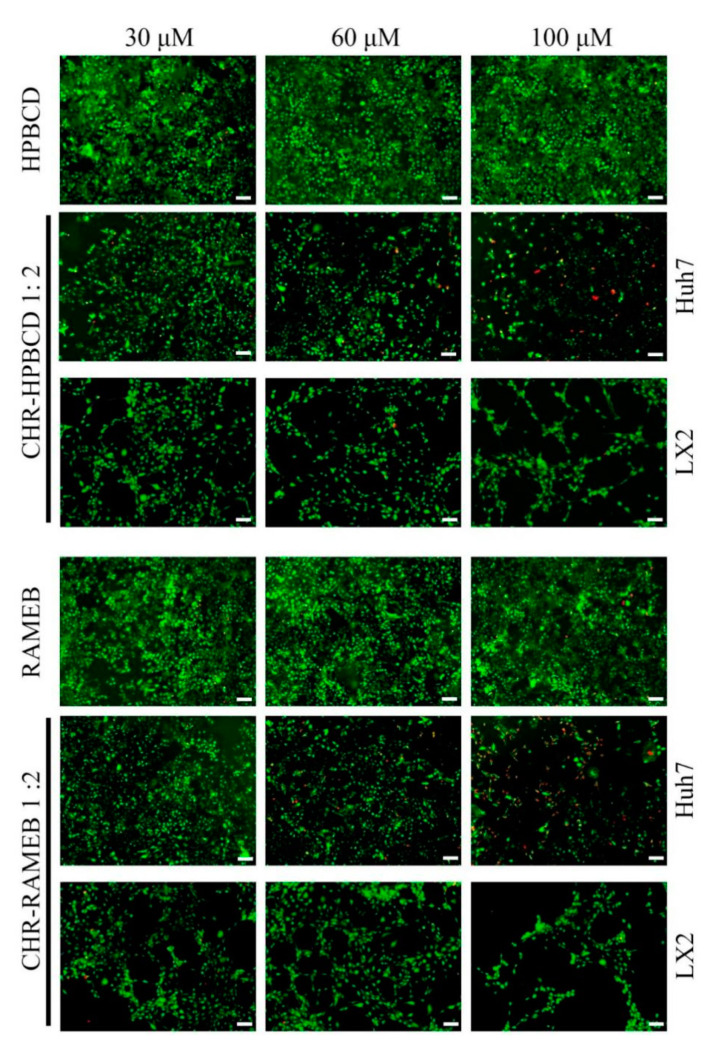
Qualitative LiveDead analysis displaying live cells (green) and nuclei of dead cells (red) of Huh7 and LX2 cells exposed to CDs (RAMEB and HPBCD) and CHR complexes (30, 60, 100 µM). Scale bar 100 μm.

The LiveDead assay showed the live cells (green) and the nuclei of the dead cells (red) in both Huh7 and LX2 cells (Figure 3) exposed to CDs (HPBCD and RAMEB) and their CHR complexes. Based on the quantitative results of MTT and LDH tests, only three representative concentrations were chosen for this test (30, 60, 100 µM) and 1:2 CD–CHR, representative of both CD–CHR complexes (Huh7), and CD–CHR 1:2, representative of both CHR–CD complexes and CDs alone (LX2) (Figure 3).

HPBCD and RAMEB showed no significant difference in the number of Huh7 live cells between the three concentrations used. In contrast, even though 30 µM 1:2 CD–CHR did not strongly affect the cells, starting with 60 µM, the number of live cells was significantly reduced and the number of dead cells was highest when exposed to 100 µM of complexes, especially for 1:2 CHR–RAMEB. These results are in accordance with the quantitative MTT and LDH results, confirming the release of CHR from the complexes.

As for HSCs, 30 µM complexed CHR with RAMEB and HPBCD, did not strongly affect the cells. However, when exposed to 60 µM CHR–CD, they showed reduced number of live cells compared to 30 µM CHR–CD. In addition, their normal cellular distribution was also affected by the presence of the complexes, and they showed a tendency to form aggregates. This tendency was best observed in the case of exposure to 100 µM CHR–CD, in which cells tended to form aggregates and detached from the culture surface, causing their further death. As determined by quantitative tests, this qualitative assay confirmed the previous results and indicated that increasing the concentration by more than 100 µM of complexes could affect the normal cells too much.

### 3.3. Analysis of NF-κB Nuclear Translocation

**Figure 4:**
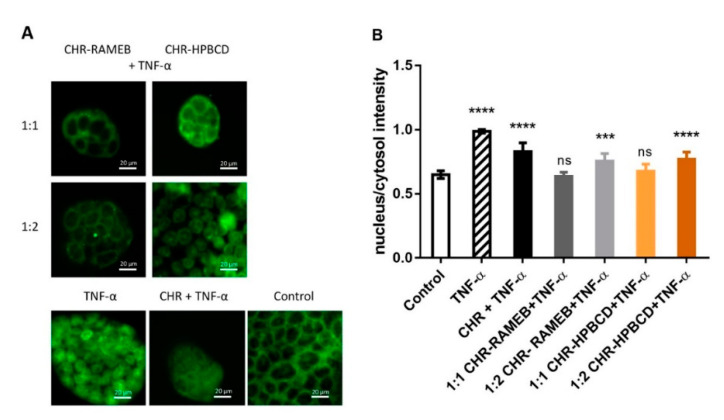
Immunohistochemical staining and analysis of NF-κB activation in CHR or CHR–CD complex-pretreated and TNF-α stimulated Caco-2 cells. (**A**) Caco-2 cells were pretreated with CHR or CHR–CD complexes and stimulated with TNF-α. Control cells were kept in culture medium. Nuclear localization of the NF-κB p65 subunit was monitored by immunostaining. Cell nuclei were labeled with Hoechst 33,342. Green: p65 staining; blue: cell nuclei. Scale bar: 20 μm. (**B**) Ratio of the fluorescence intensity of the NF-κB immunostaining in cell nuclei and cytoplasm. Values were expressed as means ± S.D., *n* = 8–10, **** *p* < 0.0001, *** *p* < 0.001, the values were compared to the value of untreated control.

The translocation of NF-κB p65 subunit from the cytosol to cell nuclei after TNF-α stimulation with or without CHR and CHR–CD complex pretreatments was detected by antibody labelling in Caco-2 cells (Figure 4). In control cells the p65 subunit is localized in the cytosol showing low nucleus/cytosol fluorescence intensity ratio, while TNF-α stimulation increased this value significantly (*p* < 0.0001). After CHR pretreatment the p65 translocation to the cell nucleus remained significantly high, compared to the control (*p* < 0.0001), however 1:1 CHR–RAMEB pretreatment avoided p65 translocation, there was no significant difference compared to the untreated cells. Similar enhanced effect could be observed in the case of 1:1 CHR–HPBCD pretreatment, while 1:2 CHR–CD complexes were less effective. It should be noted, that each pretreatment significantly decreased the p65 translocation, compared to TNF-α stimulation (*p* < 0.01).

### 3.4. Analysis of Antioxidant Effects of Chrysin and Its Cyclodextrin Complexes

**Figure 5:**
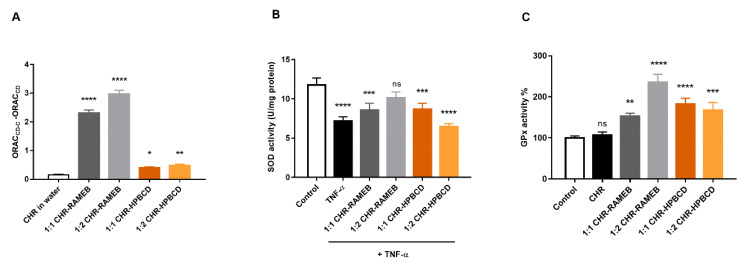
The effect of CHR and CHR–CD complexes measured in ORAC (**A**), SOD activity (**B**) and GPx (**C**) assays. Values were expressed as means ± S.D., *n* = 8–10, **** *p* < 0.0001, *** *p* < 0.001, ** *p* < 0.01, * *p* < 0.05, ns—non-significant. Values were compared to the value of CHR in water in the case of ORAC assay and to the untreated control in other assays.

Each CHR–CD complex showed significant differences in ORAC assay compared to chrysin (*p* < 0.05) (Figure 5). RAMEB complexes showed more pronounced effects than HPBCD complexes and analysing the concentration dependence showed that increasing the ratio of CD in complexes resulted in increased ORAC values. In the case of SOD activity measurement, TNF-α stimulation was significantly reduced, while CHR–cyclodextrin complexes improved the SOD activity of cells. The 1:2 CHR–RAMEB complex was the most effective. There was no significant difference between the SOD activity of 1:2 CHR–RAMEB complex-treated and control cells. The Glutathione Peroxidase (GPx) assay showed a similar tendency. The 1:2 CHR–RAMEB complex had the highest stimulation on GPx activity, but all of the CHR–CD complexes increased GPx activity significantly.

### 3.5. Analysis of α-sma Expression in LX2 Cells after Exposure to Chrysin Complexes

**Figure 6:**
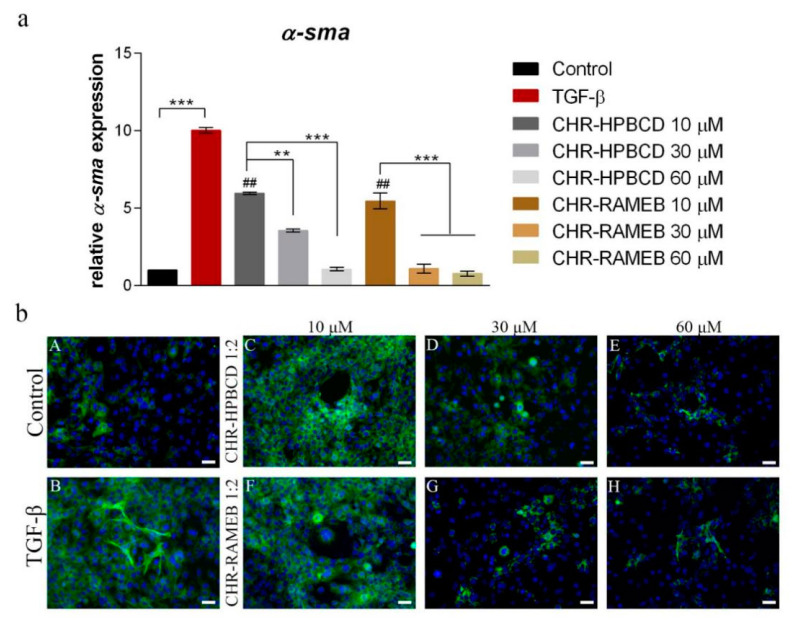
Evaluation of *α-sma* expression in HSCs after 48 h exposure to CHR complexed with RAMEB and HPBCD (1:2). (**a**) Relative gene expression of *α-sma* in HSCs. Statistical significance: ** *p* < 0.01; *** *p* < 0.001; ## *p* < 0.01 (CHR–RAMEB/CHR–HPBCD vs. TGF-β); (**b**) Immunofluorescence staining of *α-sma* (antibody conjugated with FITC, green), nuclei of the cells stained with Hoechst 33,342 (blue), (A) control; (B) TGF-β activated HSCs; (C–E) HSCs after exposure to 1:2 CHR–HPBCD 10, 30, 60 µM; (F-H) HSCs after exposure to 1:2 CHR–RAMEB 10, 30, 60 µM; scale bar 50 μm.

The expression of fibrosis marker *α-sma* was evaluated at the gene and protein levels in TGF-β activated HSCs exposed for 48 h to CHR–RAMEB/-HPBCD 10, 30 and 60 µM (Figure 6).

#### 3.5.1. Real Time PCR

Figure 6a shows the relative gene expression levels of *α-sma* in HSCs. Compared to control, cells activated with TGF-β expressed statistically significant (*p* < 0.001) higher levels (~10×) of *α-sma*, indicating that HSCs responded to TGF-β treatment and induced *α-sma* production. Exposure to CD–CHR complexes, even at the smallest 10 µM concentration, reduced the *α-sma* relative expression in HSCs by ~50%, suggesting the efficiency of CDs to release CHR, and also the anti-fibrotic potential of CHR, even in small quantities. CHR–RAMEB 30 and 60 µM statistically significantly reduced (*p* < 0.001) the expression levels of *α-sma* in HSCs compared to 1:2 CHR–RAMEB 10 µM, suggesting the efficiency of RAMEB to release CHR at smaller concentrations. CHR–HPBCD 30 µM induced statistically significant (*p* < 0.01) lower *α-sma* expression compared to 10 µM, suggesting the potential of this complex in increasing the bioavailability of CHR that regulates *α-sma* expression. Furthermore, CHR–HPBCD 60 µM reduced *α-sma* expression even more, with similar levels to control, indicating the potential of these complexes in antifibrotic treatments. All tested complexes, at 60 µM concentrations, reduced the expression of *α-sma* to control levels, but only 1:2 CHR–RAMEB induced the same response in 30 µM, indicating its potential future use at this concentration of complexed CHR.

#### 3.5.2. Immunofluorescence Staining

The results of *α-sma* gene expression were confirmed at protein level as well, by immunofluorescence staining (Figure 6b). As shown in Figure 6 (bA,bB), when activated with TGF-β, HSCs expressed high levels of *α-sma* compared to control, with cells that presented elongated fibers and exhibited myofibroblast-*like* phenotype, in accordance with gene expression levels. When exposed to CHR complexed with RAMEB/HPBCD 10 µM, the cells lost their myofibroblast-*like* phenotype, but they still expressed high levels of *α-sma* (Figure 6(bC,bF)). As the concentration was increased, *α-sma* expression was reduced, suggesting the efficiency of the complexes to release CHR and the antifibrotic potential of CHR dependent on concentration. The lowest levels of *α-sma* were obtained when activated HSCs were exposed to the highest concentration of 60 µM of either HPBCD or RAMEB complexed with CHR. However, when exposed to 30 µM of CHR–CD complexes, CHR–RAMEB was more efficient in reducing *α-sma* expression compared to CHR–HPBCD.

## 4. Discussion

Liver fibrosis represents a growing threat around the world, a condition that could be reversed under certain conditions, but that can also progress to cirrhosis or hepatocellular carcinoma, which induce irreversible liver damage. Hepatic stellate cells are the key regulators of liver fibrosis development, as they activate and produce collagen type I, the main component of ECM. Potential treatments such as the flavonoid CHR show anti-fibrotic potential, but its use is limited because of its low solubility. Complexation with CDs such as HPBCD and RAMEB could improve CHR solubility and bioavailability and enhance its effect on liver fibrosis. Based on our previous study [20], RAMEB and HPBCD were the CDs selected for in vitro biocompatibility assessment and evaluation of their anti-inflammatory, antioxidant and anti-fibrotic potential, in the present study.

The main concern in using this type of treatment approach is represented by the presence of CDs in the CHR complexes, as they could induce toxicity at certain concentrations [21]. However, the low toxicity of both complexes (HPBCD and RAMEB) used in this study was confirmed by other studies as well [22]. The low toxicity of HPBCD-based complexes was demonstrated by a number of studies presented in a review by Gould and Scott, many of them tested in vivo on animal models and patients [23]. In addition, the low toxicity induced by RAMEB was shown in another study [24], in accordance with our results that show good biocompatibility of the complexes between 10 and 60 µM on Huh7 and LX2 cells. The anti-cancer effect of CHR is well-known and an extensive validated property by a great number of studies [25,26,27]. Therefore, our study evaluated the effect of CHR from CD complexes on a hepatocellular carcinoma cell line Huh7 to assess the release of CHR between 1:1 and 1:2 complexes. CHR was efficiently released from the complexes, both from HPBCD and RAMEB, starting from 30 µM, as shown from lower cell viability levels compared to control and 10 µM. As the target cells of this potential treatment are HSCs, we exposed them to the complexes as well and evaluated their response. Cell viability is reduced in the complexes’ presence, but taken together with the cytotoxicity results, CHR complexes with CDs could be used in 10–60 µM. The 1:2 complexes proved to promote CHR release better than the 1:1 complexes. This study confirmed the biocompatibility of the investigated complexes based on CHR with RAMEB and HPBCD, which justifies the potential use of them in further studies to evaluate their anti-fibrotic potential.

We used Caco-2 intestinal cells to investigate the first event after chrysin absorption from the gastrointestinal tract. After absorption on Caco-2 intestinal cells, CD complexed CHR showed significantly higher anti-inflammatory and antioxidant effects than the non-complexed chrysin. Anti-inflammatory effect was investigated by the inhibition of the nuclear translocation of the NF-κB p65 subunit. Both CHR–RAMEB and CHR–HPBCD complexes efficiently inhibited this pathway at a 1:1 molar ratio. Interestingly, in antioxidant ORAC, SOD and GPx assays, the 1:2 RAMEB complex was the most effective. This is in accordance with our earlier results showing RAMEB to be the most potent solubilizer of CHR among the investigated CDs [20]. Further experiments focused on the effects of CHR–CD complexes on hepatic cells.

Based on biocompatibility results, the anti-fibrotic potential of CHR complexes was evaluated at three concentrations (10, 30 and 60 µM) by assessing the expression level of the HSC activation marker *α-sma*. Expression of this marker represents an early indicator of HSCs activation and is one of the most frequently used and reliable markers to indicate liver fibrosis progress [28]. Both complexes statistically significantly reduced *α-sma* expression both at the protein and gene levels, especially at 60 µM concentration, suggesting the efficient release of CHR from the complexes and its potential antifibrotic effect. However, CHR complexed with RAMEB induced a significant effect at a smaller concentration as well (30 µM) similar to the effect induced by 60 µM, suggesting RAMEB to be a better CD than HPBCD in releasing CHR. The favorable release of a flavonoid (silymarin) from RAMEB and HPBCD complexes was demonstrated in another study as well, on CCl_4_-liver fibrosis induced mice [17]. The hepatoprotective effect of CHR against numerous toxic agents was confirmed in many studies [11,12,29,30], but the anti-fibrotic effect on liver fibrosis has only recently started to gain interest. There are two studies that evaluated the potential of this effect on CCl_4_-liver fibrosis induced mice, by assessing the impact on TGF-β/Smad pathway [9] and on the TIMP/MMP balance [14].

## 5. Conclusions

In conclusion, the complexes CHR–RAMEB and CHR–HPBCD proved to be biocompatible, and showed anti-inflammatory, antioxidant and anti-fibrotic effects. The 1:2 CHR–CD complexes were more efficient in releasing CHR that further exerted its beneficial properties, and the favorable results were obtained for RAMEB complexes. To confirm its potential use in liver fibrosis therapies, 1:2 CHR–RAMEB could be used for further in vivo studies.

## Figures and Tables

**Table 1:** Characteristics of chrysin–cyclodextrin complexes measured by dynamic light scattering.

Complexes	Peak 1 Average Diameter (nm)	Peak 1 Average Area Intensity (%)	Peak 2 Average Diameter (nm)	Peak 2 Average Area Intensity (%)	Zeta-Potential (mV)
CHR–RAMEB 1:1	1.5 ± 0.1	66 ± 24	259	32 ± 21	−13 ± 1
CHR–RAMEB 1:2	1.5 ± 0.2	89 ± 15	144	22	−10 ± 1
CHR–HPBCD 1:1	2 ± 0.2	25 ± 5	206 ± 85	72 ± 4	−13 ± 2
CHR–HPBCD 1:2	2 ± 0.2	8 ± 2	143 ± 64	90.5 ± 2	−12 ± 0.3
